# An Efficient Ambient‐Moisture–Driven Wearable Electrical Power Generator

**DOI:** 10.1002/advs.202300750

**Published:** 2023-05-18

**Authors:** Debasis Maity, Martin Fussenegger

**Affiliations:** ^1^ Department of Biosystems Science and Engineering ETH Zurich Mattenstrasse 26 Basel CH‐4058 Switzerland; ^2^ Faculty of Science University of Basel Mattenstrasse 26 Basel CH‐4058 Switzerland

**Keywords:** breath power, breathing pattern analysis, electrical power generation

## Abstract

Existing devices for generating electrical power from water vapor in ambient air require high levels of relative humidity (RH), cannot operate for prolonged periods, and provide insufficient output for most practical applications. Here a heterogeneous moisture‐driven electrical power generator (MODEG) is developed in the form of a free‐standing bilayer of polyelectrolyte films, one consisting of a hygroscopic matrix of graphene oxide(GO)/polyaniline(PANI) [(GO)PANI] and the other consisting of poly(diallyldimethylammonium chloride)(PDDA)‐modified fluorinated Nafion (F‐Nafion (PDDA)). One MODEG unit (1 cm^2^) can deliver a stable open‐circuit output of 0.9 V at 8 µA for more than 10 h with a matching external load. The device works over a wide range of temperature (−20 to +50 °C) and relative humidity (30% to 95% RH). It is shown that series and parallel combinations of MODEG units can directly supply sufficient power to drive commercial electronic devices such as light bulbs, supercapacitors, circuit boards, and screen displays. The (GO)PANI:F‐Nafion (PDDA) hybrid film is embedded in a mask to harvest the energy from exhaled water vapor in human breath under real‐life conditions. The device could consistently generate 450–600 mV during usual breathing, and provides sufficient power to drive medical devices, wearables, and emergency communication.

## Graphical abstract

A heterogeneous hybrid film based on graphene oxide/polyaniline:poly(diallyldimethylammonium chloride)‐modified fluorinated Nafion is designed and a moisture‐driven electrical power generator (MODEG) is assembled. MODEGs can deliver a stable open‐circuit output of 0.9 V at 8 µA for more than 10 h with a matching external load.

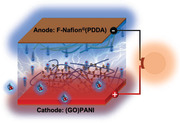

## 1 Introduction

Various principles, including streaming potential,^[^ ^1^ ^]^ ion gradients,^[^ ^2^ ^]^ triboelectrification,^[^ ^3^, ^4^ ^]^ and ion‐charge transport,^[^ ^5^ ^]^ have been utilized to generate electricity by converting the chemical potential energy of water vapor to electrical energy.^[^ ^6^, ^7^, ^8^, ^9^ ^]^ However, most devices based on these approaches generate less than 0.5 V and provide a low output current (<10 µA cm^−2^) even at high relative humidity (RH), which is insufficient to drive commercial electronics, typically requiring output currents over 1 mA cm^−2^.^[^ ^10^, ^11^, ^12^ ^]^ Therefore, there is a great need for robust, scalable high‐performance devices able to directly power medical devices and wearables, as well to provide emergency illumination and communication in situations where other systems, such as solar‐powered devices, cannot function.^[^ ^1^, ^13^ ^]^

**Figure 1:**
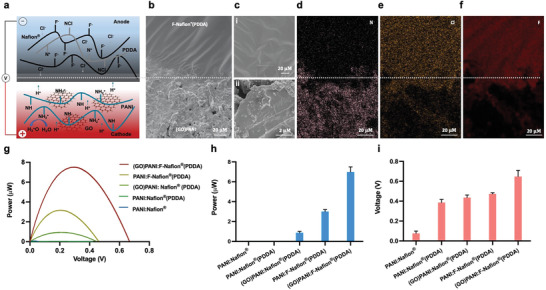
Working principle, structure, and bilayer optimization of MODEG. a) Schematic illustration of moisture‐enabled electrical power generation in the (GO)PANI:F‐Nafion (PDDA) bilayer. The bilayer enables spontaneous water adsorption from air and ion dissociation in humid air. The Cl^−^ ions of the F‐Nafion (PDDA) layer and the H^+^ ions of the (GO)PANI layer diffuse in opposite directions thereby producing two local transmembrane potentials that generate the electrical power in the MODEG. b) Field emission scanning electron microscopy (FESEM) image of the (GO)PANI:F‐Nafion (PDDA) film showing the F‐Nafion (PDDA) anode layer at the top (above the dotted line) and the (GO)PANI cathode layer at the bottom (below the dotted line). c) Enlarged FESEM images of the F‐Nafion (PDDA) anode surface (i) and the (GO)PANI cathode surface (ii). Element mapping of d) nitrogen, e) chlorine, and f) fluorine in the (GO)PANI:F‐Nafion (PDDA) film shown in (b). In panels (b,d–f), the top part of the image is the F‐Nafion (PDDA) region and the bottom part is the (GO)PANI region. g–i) Output power dynamics (g), output power (h) and voltage (i) of MODEGs containing different modified bilayer materials at 83% relative humidity. Bars represent mean ± SEM (*n* = 5).

Inspired by the ability of the mitochondrial enzyme ATP synthase to catalyze the synthesis of ATP as a cellular energy source driven by a proton gradient,^[^ ^14^ ^]^ we designed a heterogeneous moisture‐driven electrical power generator (MODEG) that employs a hybrid bilayer system. As shown in **Figure** **1**a, one layer consists of a hygroscopic matrix of graphene oxide/polyaniline [(GO)PANI],^[^ ^13^ ^]^ and the other consists of poly(diallyldimethylammonium chloride) (PDDA)‐modified fluorinated Nafion [F‐Nafion (PDDA)]. In this system, polyaniline (PANI) acts as hygroscopic material to capture water from the ambient environment and produce H^+^ ions that are readily transported via the graphene oxide (GO) component to generate a proton concentration gradient.^[^ ^11^ ^]^ Thus, (GO)PANI serves as a cathode layer. In contrast, fluorinated Nafion is a proton‐selective material, allowing free Cl^−^ ions to accumulate in the poly(diallyldimethylammonium chloride (PDDA) component, and this layer serves as the anode side of the (GO)PANI:F‐Nafion (PDDA) bilayer. The highly conductive anode and cathode layers produce a potential difference owing to oppositely directed diffusion of positively charged H^+^ ions from the (GO)PANI layer and negatively charged Cl^−^ ions from the F‐Nafion (PDDA) layer.^[^ ^15^, ^16^ ^]^

We show here that series and parallel combinations of our MODEG units can provide sufficient power to drive commercial electronic devices. To illustrate the practical value of our system, we embedded (GO)PANI:F‐Nafion (PDDA) hybrid film in a mask; this setup consistently generated 450–600 mV from exhaled water vapor during usual breathing, and provided sufficient power for practical applications.

## 2 Results

### 2.1 Design, Construction, and Validation of MODEG Materials

The MODEG device consists of a hygroscopic matrix of (GO)PANI upon which F‐Nafion (PDDA) is sprayed to generate a free‐standing bilayer‐hybrid film (Figure 1a). Water molecules interact with the charged sites of GO(PANI) and H‐bond–accepting or ‐donating sites of PANI (=NH^+^, —NH_2_^+^), dissociating into protons (H^+^)/H_3_O^+^ and hydroxyl ions (OH^−^). The water molecules absorbed in the PANI matrix act as a source of protons and conduct ion charge through the GO(PANI) network.^[^ ^17^ ^]^ GO shows excellent conductivity, promoting interlayer ion transport across the GO(PANI) composite. Nafion is a proton‐selective layer and provides a platform for H^+^ transport, while modified fluorinated Nafion (F‐Nafion ) exhibits enhanced electronegativity and enhanced driving force for proton transport.^[^ ^16^ ^]^ PDDA serves as a source of mobile electronegative Cl^−^ ions. Thus, the (GO)PANI composite generates the positive charge carrier (H^+^) and forms the cathode, while the F‐Nafion (PDDA) component provides free negative ions (Cl^−^) and forms the anode part of the MODEG.^[^ ^5^ ^]^

Field emission scanning electron microscopy (FESEM) shows that the final bilayer consists of GO:PANI and F‐Nafion (PDDA) aggregates (Figure 1b,c). The nanochannels between (GO)PANI and F‐Nafion (PDDA) are sufficiently wide to permit the permeation and diffusion of water and ions. Elemental analysis confirmed the presence of nitrogen atoms, reflecting the location of positively charged —NH_2_^+^ groups, on the (GO)PANI side, as well as negatively charged Cl^−^ and F^−^ groups on the F‐Nafion (PDDA) side of the bilayer, as expected (Figure 1d–f).

In the presence of moisture in the measurement chamber (Figure 1g), 1 cm^2^ of the (GO)PANI:F‐Nafion (PDDA) bilayer generates a stable maximum open circuit voltage of 0.67 V at 83% relative humidity (Figure 1g). Furthermore, characterization of the maximum power output and voltage of various bilayer materials confirmed that (GO)PANI:F‐Nafion (PDDA) indeed showed the best performance (Figure 1h,i). The asymmetrical metal electrode connection with the GO composite forms a Schottky contact, which promotes unidirectional transport of mobile ions, greatly enhancing the power output of the device.^[^ ^18^ ^]^

### 2.2 Effect of Humidity on MODEG Performance

**Figure 2:**
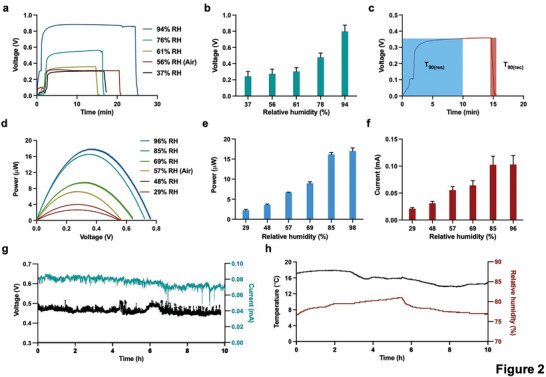
Characterization of electrical power generation by MODEGs. a,b) Time course (a) and absolute values (b) of open circuit voltage at different levels of relative humidity (RH). c) MODEG response and recovery times. Blue shading shows the response time to reach a saturation voltage of 90% in the presence of 94% relative humidity (*T*_90(res)_) and brown shading indicates the time required to decrease saturation voltage by 90% in the presence of dry air. d) Relationship between MODEG output power and voltage at different levels of relative humidity (RH). e,f) Comparisons of e) maximum power and f) short circuit current at different levels of relative humidity (RH). g,h) Voltage output (black line) and resulting current (green line) of the MODEGs operating for 12 h under atmospheric conditions (g) at the indicated recorded temperature (black line) and relative humidity (brown line) (h). Bars represent mean ± SEM (*n* = 3).

The effect of relative humidity (RH) on the performance of the MODEG is shown in **Figure** **2**. The open circuit voltage (*V*_OC_) increased with increasing RH (Figure 2a,b), reached a maximum value of ≈870 mV within 3 min and decreased rapidly when air was evacuated from the system (Figure 2c). This represents an up to six times faster reaction time compared to previous systems.^[^ ^19^ ^]^ The output power and voltage at various levels of RH are shown in Figure 2d–f. The MODEG device could consistently generate a DC voltage output of at least 0.48 V and a current of 0.073 mA for more than 10 h in an ambient environment (Figure 2g) with fluctuating temperature and relative humidity (Figure 2h).

### 2.3 MODEG Performance Optimization

**Figure 3:**
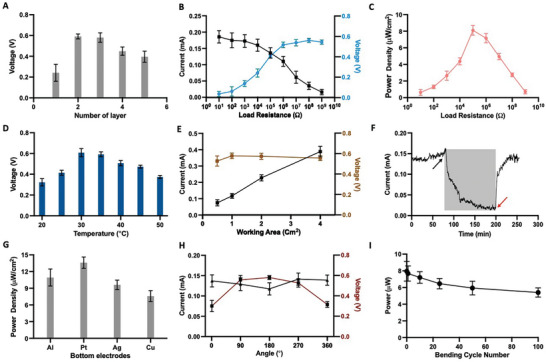
Optimization of MODEGs. a) Relationship between output voltage and number of F‐Nafion (PDDA) layers. b) Output current (black line) and voltage (green line) in response to increasing electronic load resistance. c) Relationship between power density and electronic load resistance. d) The output voltage of MODEG at different working temperatures. e) Output current (black line) and voltage (blue line) as a function MODEG surface area. f) Effect of atmospheric conditions on MODEG current. Air was first removed from a MODEG‐containing chamber by a vacuum pump (black arrow, gray shade), followed by blowing in humidified air (94% relative humidity). g) Output power of MODEGs containing different bottom electrode materials. h) Impact of forward and backward bending at various angles on the current (black line) and output voltage (brown line) of MODEGs. i) Power output of MODEGs after increasing numbers of bending cycles at 180°. Bars represent mean ± SEM (*n* = 3).

Further investigation established that the electric output is dependent on many factors, including the working electrode area, number of spray layers, external load, and environmental conditions including ambient temperature and humidity. The voltage increased from 0.25 V with one spray layer to over 0.6 V with three spray layers, but then decreased with further increase in the number of spray layers (**Figure** **3**a). With a single spray layer, the MODEG is very thin and the ion concentration gradient is small, so that the output voltage is low. With more than three spray layers, the MODEG becomes thick, and the internal water diffusion and ion transport pathways become longer, resulting in a reduction of the output voltage.^[^ ^12^ ^]^ We also measured the voltage and current as a function of external load resistance in the range of 10 to 10^9^ Ω (Figure 3b). With increasing resistance, the output voltage increased gradually from 36 to 560 mV, whereas the output current decreased from 0.19 to 0.01 mA. The maximum power density reached 8 mW cm^−2^ with a load resistance of 10^5^ Ω (Figure 3c). In contrast, the output voltage and current of most previously reported humidity power generators are intermittent, restricting their practical utility.^[^ ^12^ ^]^ The maximum voltage and current output show low crest factors (the ratio of the peak value to the root‐mean‐square value) of 1.37 and 1.40 respectively, indicating that the MODEG exhibits good power‐generating ability over a wide range of output load.^[^ ^20^ ^]^

Next, we examined the effect of temperature in the range of −20 to +50 °C, which might cover the range of practical use. The output voltage increased to a maximum at 30 °C, and then slowly decreased with further increase of the temperature (Figure 3d). The MODEG can still facilitate proton dissociation from moisture to some extent at −20 °C, producing a low voltage of about 180 mV. At higher working temperatures, the thermal motion of water molecules and the ion transport rate increase and as a consequence the output voltage increases with increasing temperature.^[^ ^12^ ^]^ As expected, the output voltage was unaffected by the working area, but the current increased linearly with working area (Figure 3e). We also examined the effect of humidity. As the humidity was decreased from 84% to 8% RH, the current decreased dramatically, then when the humidity was restored, it fully recovered to about 0.15 mA (Figure 3f). Due to its operability across a wide range of temperatures and RH, MODEGs could be used in most weather conditions and climate zones around the globe.

We also examined the effect of different materials for the bottom electrode (Figure 3g). Various electrode materials were examined, and silver was chosen as the bottom electrode material for further application due to susceptibility of aluminum to corrosion and the high cost of platinum, despite their superior performance. The robustness of the MODEG device was also investigated by bending it at different angles and for different numbers of cycles. Bending at 180° caused a decrease of the current and an increase of the voltage (Figure 3h), but the fluctuation of output power was negligible over 100 continuous bending cycles (Figure 3i), suggesting that the device would be suitable for use in wearable devices.

### 2.4 Practical Applications of MODEGs

**Figure 4:**
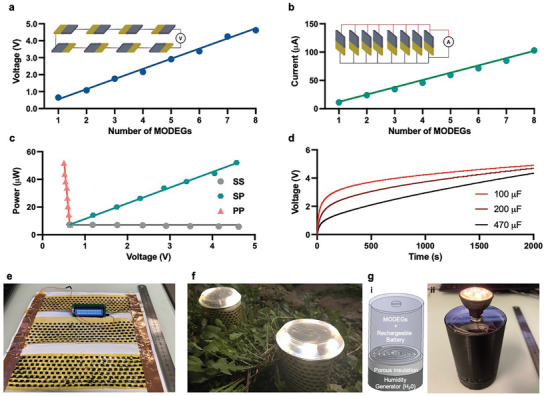
Real‐world applications of MODEGs. a) Voltage of different numbers of MODEG units connected in series. b) Current of different numbers of MODEG units connected in parallel. c) Relationship between power output and voltage of different numbers of individual MODEG units connected in series (SS), in parallel (PP), and in series as well as in parallel (SP). d) Voltage time‐course of commercial capacitors (100, 200, and 470 µF) charged by a package of MODEG units (22 in‐series‐linked strings of 24 MODEG units in parallel). e) 16 × 2 LED display module powered by an integrated 22 × 24 MODEG package connecting 24 in‐series‐linked strings of 22 MODEG units in parallel. f) Photograph of illuminated night lamps autonomously powered by a MODEG package, connecting 22 in‐series‐linked strings of 16 MODEG units in parallel, linked to a storage capacitor. g) A MODEG‐powered handheld flashlight. (i) shows a schematic of the flashlight, which contains a water chamber providing high relative humidity and an insulated MODEG package consisting of eight in‐series‐linked strings of 44 MODEG units in parallel connected to a rechargeable lithium polymer battery powering a standard 10 V GU5.3 LED bulb to provide 200 lumens. (ii) shows a photograph of the flashlight in operation.

To examine the practical scalability, we evaluated series and parallel configurations of MODEGs. The output voltage was enhanced by connection in series, as shown in **Figure** **4**a. For example, 4.9 V could be reached by connecting eight 1 cm^2^ MODEGs in series. A parallel configuration of eight devices yielded a stable current of 109 mA and voltage of 0.57 V (Figure 4b). Increasing numbers of series connections linearly increased the voltage, while increasing numbers of parallel connections linearly increased the current (Figure 4c), and sufficient power output was achieved to directly operate many common low‐power electronic and internet of things devices, such as LCD displays, application‐specific integrated circuits, field‐programmable gate arrays, and MOSFET transistors.^[^ ^21^ ^]^ The electric power supplied by scalable MODEGs is capable of charging commercial capacitors of 100, 200, and 470 µF up to 5 V using integrated 22 × 8 MODEG arrays (22 in‐series and eight parallel connections) (Figure 4d).

To validate MODEGs in real‐world proof‐of‐concept situations, we designed and tested them to power LED displays (Figure 4e) as well as LED lights (Figure 4f,g). A MODEG package consisting of 24 parallel‐connected strings of 22 in‐series MODEGs was able to autonomously power a commercially available 2 × 16 LED display for extended periods of time when connected to a power storage device such as an off‐the‐shelf rechargeable lithium polymer battery (Figure 4e). Additionally, a MODEG package containing eight parallel‐connected strings of 22 in‐series MODEGs could charge a standard rechargeable lithium polymer battery (600 mAh) during the day and illuminate a commercial garden lamp containing six LEDs during an entire night (Figure 4f). We also designed a MODEG‐powered hand‐held emergency flashlight (Figure 4g). This flashlight consists of a water container producing a high relative humidity of 93%, a porous filter insulating the MODEG against short‐circuiting, and a 44 × 8 MODEG pack (eight parallel‐connected strings of 44 in‐series MODEGs) connected to a 600 mAh rechargeable lithium polymer battery and a 10 V GU5.3 LED bulb. When the tank is filled with water, battery charging is boosted, which triggers 200‐lumen illumination by a standard 12 V GU5.3 LED bulb for extended periods of time (Figure 4g). All of these examples show that MODEG packages are scalable and can be tailored to particular electrical power needs.

### 2.5 A MODEG‐Based Breath‐Powered Device

**Figure 5:**
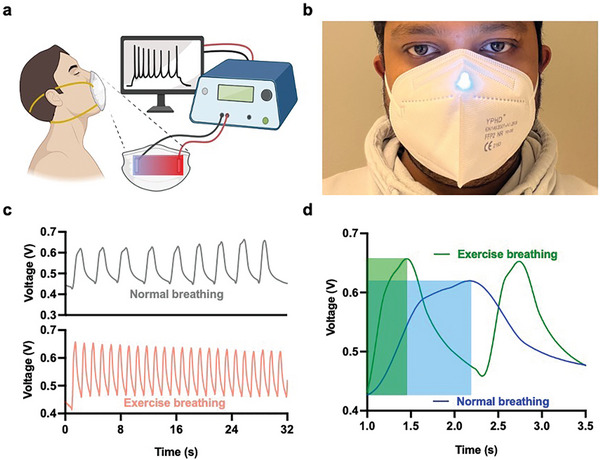
a) Schematic illustration of the use of a MODEG‐laminated face mask to generate electrical power and for real‐time monitoring of breathing. b) Patterns of voltage output of a 4 × 4 cm^2^ MODEG laminated into a commercial FDA‐licensed FFP2 face mask during normal breathing and breathing during exercise. c) Profiles of voltage output of a 4 × 4 cm^2^ MODEG laminated into a commercial FDA‐licensed FFP2 face mask during individual breaths and d) time curves for a single breathing pattern (normal and exercise breathing).

To further demonstrate the advantage of flexible (GO)PANI:F‐Nafion (PDDA)‐based MODEGs for easy integration into wearable objects, we built a wearable face mask to capture the energy from the humidity of exhaled breath and investigated the effect of different human breathing patterns (**Figure** **5**a). We embedded a 4 × 4 cm^2^ MODEG bilayer film inside a commercial FDA‐licensed FFP2 face mask (Figure 5b and Movie S1, Supporting Information). This device consistently generated up to 600 mV and 120 µA during usual breathing and up to 650 mV and 136 µA during exercise (Figure 5b and c), which would be sufficient to power and control portable medical devices (glucometers, insulin pumps, and hearing aids), wearable electronics (GPS trackers, smart watches, smart bracelets, and smart rings) as well as emergency notification and communication systems (short‐wave radio, cell phones, and satellite texting).^[^ ^22^, ^23^, ^24^, ^25^, ^26^, ^27^ ^]^ The response time to achieve maximum voltage under normal and exercise‐induced breathing was 1.3 and 0.5 s, respectively (Figure 5c and d). We also showed that the MODEG‐based breath‐powered mask could be used as a self‐powered device for monitoring human breathing patterns, as exemplified by profiling breathing after sporting activity or during inactivity (Figure 5b) (Movie S1, Supporting Information).

## 3 Conclusion

There is an increasing need for devices generating off‐grid electrical power to operate wearable electronic devices, run medical equipment, and enable emergency communication in remote places. Solar power panels are currently the gold standard for self‐sufficient electrical power generation. However, solar cells only function during daylight, are sensitive to weather conditions, and require battery packs for storage of electrical power during suboptimal conditions. This decreases the overall efficiency of solar power systems to around 50%,^[^ ^28^ ^]^ and limits or prevents their use in many climatic conditions, in particular in dark and cold regions such as the arctic. Besides solar power panels, piezoelectric,^[^ ^29^ ^]^ thermoelectric,^[^ ^30^ ^]^ triboelectric,^[^ ^31^ ^]^ electromagnetic,^[^ ^32^ ^]^ and electrochemical^[^ ^33^ ^]^ systems have been explored as autonomous self‐sufficient power generators, but they require chemical fuels (electrochemical cells), are extremely motion‐dependent (electromagnetic devices), require unphysiological thermal gradients (thermoelectrical systems), or produce insufficient power for real‐world applications (piezo‐ and triboeletric technologies).

Recently, autonomous self‐sufficient electrical power generators consisting of nanomaterials producing electrical energy from water vapor in ambient air are gathering momentum as a means to produce electrical energy at remote locations.^[^ ^1^, ^2^, ^3^, ^4^, ^5^ ^]^ However, current forefront designs require high‐level relative humidity (>30% RH) and do not provide sufficient electrical output to power and control devices for most practical applications.^[^ ^20^ ^]^ For example, the LiCl‐loaded cellulon paper system provided an electrical output of only 0.8 V at 7 µA,^[^ ^6^ ^]^ while adsorption–desorption humidity‐based power generators fabricated from sodium alginate, silicon dioxide nanofibers, and reduced graphene oxide exhibited an output power of 0.5 V at 100 µA.^[^ ^20^ ^]^ Heterogeneous devices based on graphene oxide provided a high output voltage approaching 1.5 V, but the resulting current was low (120 nA) and unstable.^[^ ^7^ ^]^ A 3D‐polypyrrole anion‐gradient material provided up to 60 mV at 86% relative humidity,^[^ ^11^ ^]^ while biological nanofiber‐based generators capitalizing on the streaming potential of absorbed water generated 120 mV,^[^ ^8^ ^]^ and titanium dioxide‐based nanowire networks reached an output power density of up to 4 µW cm^−2^ at 85% relative humidity.^[^ ^24^ ^]^ The recent combination of polyelectrolytes and phytochromes provided high performance (0.92 V, 1.55 mA cm^−2^), but most of the power was generated by light and not humidity, which negates the advantage compared to solar power panels in thin air.^[^ ^28^ ^]^

Here we present a MODEG in the form of a bilayer combining a hygroscopic matrix of (GO)PANI with F‐Nafion (PDDA) providing a polyelectrolyte layer. In the presence of moisture, this hybrid bilayer film produces spontaneous charge separation with subsequent directional movement of H^+^ ions leading to a stable output power density of 16 µW cm^−2^, an open circuit voltage of 0.9 V and a short circuit current of 126 µA. The MODEG system auto‐recycles the humidity in the atmosphere and operates over wide ranges of temperatures (−20 to +50 °C) and relative humidity (30% to 93%). Therefore, MODEGs not only provide an unmatched level of self‐sufficient power output that is compatible with most climatic conditions around the globe, but also are flexible, inexpensive, and simple to fabricate, as well as scalable both in surface size and by in‐series and/or in‐parallel connection. Already a small package containing eight parallel‐connected strings of 22 in‐series 1 cm^2^ MODEGs or a single 5 × 5 cm MODEG patch has been demonstrated to power emergency lights and hand‐held 200‐lumen flashlights as well as LED displays. Due to their flexibility and resistance to extensive bending, MODEGs are particularly suited as a wearable power source and could in principle be laminated into clothing. A textile cover would also add an additional layer of protection for the MODEG against wear and rain, while still enabling moisture to reach the power generator. MODEG‐laminated L‐size trousers and shirts with an average surface of 1 m^2^ would in principle be able to generate an output power density of ≈90 mW m^−2^ and an open circuit voltage of around 0.50 V which would be sufficient to power up consumer electronics (smart watches, web cameras, headphones, and MP3 players), digital instruments (pedometers, calculators, and digital thermometers), and medical devices (glucometers, insulin pumps, and sphygmomanometers).

As a proof‐of‐concept real‐world application we laminated 5 × 5 cm MODEGs into face masks designed to protect against airborne viral infections. Natural breathing provided a continuous source of high relative humidity that enabled the MODEG to produce enough power to operate simple LED displays, medical devices, and emergency communication. In addition to providing electrical power, MODEGs laminated into face masks could also be used to monitor the breathing activity of humans in real time to follow high‐altitude activities,^[^ ^34^, ^35^ ^]^ monitor psychological disturbances such as depression, anxiety, and stress,^[^ ^36^, ^37^ ^]^ track drug abuse,^[^ ^38^, ^39^ ^]^ or help diagnose and profile severe medical conditions including asthma, pneumonia and chronic obstructive pulmonary disease.^[^ ^27^, ^40^, ^41^, ^42^, ^43^ ^]^ Since MODEGs are very thin and flexible they could in principle also be laminated onto the dark backside of solar power panels in order to complement electrical energy production when solar panels fail to deliver: at night, in shaded situations, and in overcast weather.

We believe that MODEGs have the potential to provide autonomous electrical energy production to power wearable electronics and control portable medical devices as well as emergency communication devices under conditions in which other systems such as solar power panels and batteries may not deliver, such as at night and in cold weather conditions.

## 4 Experimental Section

### Chemicals and Materials

Graphite powder, potassium permanganate, sulfuric acid, sodium nitrate, hydrogen peroxide, ammonium persulfate, aniline, PDDA solution, Nafion 117 solution, trichloro(1H,1H,2H,2H‐perfluorooctyl)silane, hydrochloric acid, and other chemicals were purchased from Sigma‐Aldrich (Buchs, Switzerland) and used as received. Polyimide films with a thickness of 125 µm were also purchased from Sigma‐Aldrich. Silver Flake Ink (Metalon HPS‐021LV) was purchased from Novacentrix (Austin, USA). Polymer aluminum capacitors were purchased from Mouser Electronics (Munich, Germany). The Joy‐it SBC‐LCD 16 × 2 display module (HD44780) (cat. no. 1 503 825), L10 Solar Garden Light (0.2 W, SMD LEDs; cat no. 2 497 614), and Philips GU5.3 12 V/3.5 W/200 lumen LEDspot Light (Philips) were purchased from Conrad Electronics AG (Wollerau, Switzerland). The rechargeable lithium polymer battery (3.7 V/600 mAh) was purchased from RS PRO (cat. no. 176–9379; RS PRO, Wädenswil, Switzerland).

### Synthesis of Graphite Oxide

Graphite oxide (GO) powder was synthesized by the oxidation of graphite powder using a modified Hummers’ method.^[^ ^44^ ^]^ Briefly, 2 g of graphite powder and 1 g of NaNO_3_ were slowly added to 50 mL H_2_SO_4_ (98 wt%) and the mixture was stirred with a magnetic stirrer at 400 rpm for 2 h in an ice–water bath. Next, 8 g of KMnO_4_ was slowly added and stirring was continued at 400 rpm for 4 h. Then, 100 mL of deionized water was added dropwise and the mixture was stirred at 30 °C for 20 min. The reaction was stopped by adding 20 mL of H_2_O_2_ (37 wt%). The mixture was vacuum‐filtered and washed with HCl (10 wt%) and the resulting GO slurry was dispersed in deionized water and centrifuged at 5000 × *g* for 10 min. Overnight heating at 130 °C afforded the GO powder.

### Preparation of (GO)PANI

In a typical synthesis, 2 g of GO and aniline monomer (5 mL) were dissolved in distilled water (100 mL, pH 7) by sonication for 10 min at 200 kJ (sonication power) (SONOPULS mini20, BANDELIN, Schaffhausen, Switzerland) followed by stirring at 200 rpm for 2 h to obtain a uniform suspension. This suspension was further stirred at 400 rpm for 2 h to obtain a stratified system with a clear interface. Then, 2 mL of HCl (37%) was added dropwise and the mixture was agitated at 30 °C for 20 min. Oxidative polymerization of aniline was conducted by dropwise addition of ammonium persulfate (0.1 m, 10 mL). The final PANI‐grafted GO ((GO)PANI) powder was collected by centrifugation at 5000 × *g* and freeze‐dried (Freeze dryer ALPHA 1–2 LDplus, Adolf Kühner AG, Basel, Switzerland).

### Preparation of F‐Nafion (PDDA)

A mixture of 10 mL of Nafion and 200 µL of trichloro(1H,1H,2H,2H‐perfluorooctyl)silane was sonicated for 15 min and stirred for 2 h to obtain a uniform suspension of F‐Nafion. The F‐Nafion was mixed with 10 mL of poly(diallyl dimethylammonium chloride) solution and stirred at 100 rpm for 6 h at room temperature. The mixture was further sonicated for 5 min at 150 kJ (SONOPULS mini20, BANDELIN, Schaffhausen, Switzerland) with a 5 s on/off cycle to obtain a uniform suspension of F‐Nafion (PDDA).

### Fabrication of GO(PANI):F‐Nafion (PDDA) Bilayer Film and Integrated Devices

First, 100 mL of ethanol and 1 g of ((GO)PANI) powder (1 v%) were mixed to prepare a homogeneous solution. The solution was sparingly spray‐coated onto a polyamide membrane containing screen‐printed silver electrodes and copper wire connections (KW30YL‐15 M, 1 × 0.05 mm^2^, Conrad Electronics AG, Wolerau, Switzerland) to fabricate the cationic layer of the bilayer. Similarly, the anion layer was formed by spray‐coating F‐Nafion (PDDA) (10 v%) between the cationic layer and the silver electrode using a PRO‐TEK dual‐action gravity air brush (PRO‐TEK, Longueuil, Canada) set to 15 Psi air pressure, 40 mm s^−1^ sweep speed, 2.5 mL min^−1^ flow rate and a substrate‐to‐nozzle distance of 15 cm. The final thickness was controlled by varying the number of spray layers, and the size was controlled by the masked area during spraying over the polyamide film. The size of a standard MODEG was set to 1, 2, or 5 cm^2^ with an average film thickness of about 100 µm. The series and parallel connections between individual MODEGs were made with prefabricated screen‐printed silver electrodes.

### Electrical Output Measurements and Characterization

Open‐circuit voltage (OCV) and short‐circuit current were measured with a potentiostat (CHI 760, CH Instruments Inc., Austin, USA). The voltage and current were recorded in real time using a Keithley 2636B source monitor (Tektronix, Beaverton, USA). The circuit parameter of the OCV test was set to a current of 0 nA and the short‐circuit current test was set to a voltage of 0 V. The humidity and temperature were measured with a data logger (RS PRO 1365, RS Components Ltd., Northamptonshire, UK). The morphology and composition of MODEGs were validated by FESEM. Elemental mapping and analysis were done by energy‐dispersive X‐ray spectroscopy using a Zeiss GeminiSEM 450 (ZEISS, Oberkochen, Germany). The RH in the test chamber was controlled by using different saturated salt solutions and monitored with a humidity sensor (RS PRO 1365, RS Components Ltd., Northamptonshire, UK). In this manuscript, the proof‐of concept testing (only the lead author himself was involved) was not subject to ethics approval either by the Swiss Cantonal Ethics Commission or by the ETH Zurich Ethics Commission.

### Statistical Analysis

All data sets were analyzed by two‐way ANOVA using GraphPad Prism software (version 8.4.3, La Jolla, CA, USA). Data are presented as mean ± standard deviation (SD) for *n* = 3 or *n* = 5, as noted in the figure legends.

## Conflict of Interest

The authors declare no competing financial interests.

## Author Contributions

D.M. and M.F. designed the project, analyzed the results, and wrote the manuscript.

## Supporting information

Supporting Information

## Data Availability

The data that support the findings of this study are available from the corresponding author upon reasonable request.
